# Artificial Intelligence Holds Promise for Transforming Public Health Nutrition

**DOI:** 10.3390/nu16234034

**Published:** 2024-11-25

**Authors:** Ruopeng An, Yuanyuan Yang

**Affiliations:** 1 Constance and Martin Silver Center on Data Science and Social Equity, New York University Silver School of Social Work, New York, NY 10003, USA; ruopeng.an@nyu.edu; 2 Brown School, Washington University in St. Louis, St. Louis, MO 63130, USA

The intersection of artificial intelligence (AI) and public health nutrition is rapidly evolving, offering transformative potential for how we understand, assess, and improve population health. This Special Issue gathers together a collection of six pioneering studies that exemplify the diverse and impactful roles AI can play in this field. From policy analysis to predictive analytics, the included research reflects a growing recognition of AI’s capacity to revolutionize how we address public health challenges and implement nutrition-related interventions.

Among the studies featured in this Special Issue, two stand out for their focus on AI-enhanced policy analysis and regulatory support. Yang et al. [1] employ natural language processing techniques to assess public sentiment regarding menu labeling regulations in the United States. Their analysis reveals a shift toward neutral sentiments following key regulatory milestones and underscores AI’s ability to monitor public engagement with health policies, offering valuable insights for policymakers aiming to refine regulatory strategies. Similarly, Hu et al. [2] utilize transformer-based models to automate the classification of food processing levels across multiple countries. Their model provides a robust tool for policymakers to track and regulate ultra-processed foods, supporting public health objectives globally.

AI’s predictive power is another prominent theme in this Special Issue, with several studies demonstrating its potential to enhance health monitoring and clinical decision-making. Martin-Morales et al. [3] present a model that predicts cardiovascular disease mortality by integrating dietary and health data, offering a powerful tool for preventive care strategies. Ren et al. [4] further illustrate AI’s predictive capabilities in a clinical setting, where their model noticeably reduces prediction errors for complications and hospitalization durations, optimizing hospital resource management. Additionally, Wu et al. [5] showcase an AI application for assessing infant stool consistency, outperforming traditional methods and providing crucial support for mothers, particularly those at risk of postpartum depression.

The scoping review by Sosa-Holwerda et al. [6] provides a comprehensive synthesis of AI’s applications in nutrition research, mapping out key areas of impact such as dietary assessment and malnutrition prediction. The review highlights both the opportunities and challenges AI presents, particularly in less explored domains like lifestyle interventions and the nuanced understanding of diet-related diseases. The authors call for further exploration in these areas to fully harness AI’s potential for transforming public health nutrition.

Three overarching trends emerge from the studies included in this Special Issue. First, the use of AI in public health policy analysis is a critical area of focus. By leveraging advanced natural language processing and machine learning techniques, the studies demonstrate AI’s capability to capture and analyze public sentiment, support regulatory efforts, and facilitate evidence-based policymaking. Second, AI’s role in predictive analytics is highlighted as a key factor in improving health outcomes. The integration of vast arrays of health and dietary data enables more accurate forecasts of disease outcomes and the optimization of healthcare resources, paving the way for more personalized and effective health interventions. Third, while AI offers significant advancements, challenges such as data bias, privacy concerns, and the need for human oversight remain crucial. Addressing these challenges is essential to ensuring that AI-driven solutions are ethical, reliable, and beneficial for all populations.

In conclusion, this Special Issue offers a deep dive into the evolving role of AI in this critical field. The studies collectively underscore the transformative potential of AI in enhancing public health nutrition through policy analysis, regulatory practices, and health monitoring. However, as we advance, it is imperative to balance innovation with ethical considerations, ensuring that AI applications are equitable, transparent, and grounded in robust validation. Future research should build on these findings, exploring the complex interplay between AI-driven and traditional health strategies. Collaborative efforts across disciplines and sectors will be vital in harnessing AI’s full potential to improve public health nutrition, ultimately leading to better health outcomes for diverse populations worldwide.
